# Temporal Genetic Dynamics of an Invasive Species, *Frankliniella occidentalis* (Pergande), in an Early Phase of Establishment

**DOI:** 10.1038/srep11877

**Published:** 2015-07-03

**Authors:** Xian-Ming Yang, Heng Lou, Jing-Tao Sun, Yi-Ming Zhu, Xiao-Feng Xue, Xiao-Yue Hong

**Affiliations:** 1 Department of Entomology, Nanjing Agricultural University, Nanjing, Jiangsu 210095, China

## Abstract

Many species can successfully colonize new areas despite their propagules having low genetic variation. We assessed whether the decreased genetic diversity could result in temporal fluctuations of genetic parameters of the new populations of an invasive species, western flower thrips, *Frankliniella occidentalis*, using mitochondrial and microsatellite markers. This study was conducted in eight localities from four climate regions in China, where *F. occidentalis* was introduced in the year 2000 and had lower genetic diversity than its native populations. We also tested the level of genetic differentiation in these introduced populations. The genetic diversity of the samples at different years in the same locality was not significantly different from each other in most localities. *F*_ST_ and STRUCTURE analysis also showed that most temporal population comparisons from the same sites were not significantly differentiated. Our results showed that the invasive populations of *F. occidentalis* in China can maintain temporal stability in genetic composition at an early phase of establishment despite having lower genetic diversity than in their native range.

There is mounting evidence that species can successfully colonize new areas disjunct from their original habitat despite their propagules having low genetic variation^1^,^2^,^3^. Low genetic diversity in invasive populations can result from genetic drift, bottlenecks, founder effects or bridgehead effects, the latter which refers to that an invasive population is the source for further invaded areas^4^,^5^. Low genetic diversity could also result from other factors, including positive selection or anthropogenic control^6^. The decreased genetic diversity can limit the adaptive ability and fitness of the newly founded individuals because of the possible loss of alleles corresponding to ecologically important traits on which selection pressures can act^7^,^8^,^9^. Yet, to our knowledge, the temporal genetic dynamics of newly established populations have not been thoroughly investigated. Such investigations could help to better understand bioinvasions and rapid evolution and lead to new strategies for pest management.

The invasive western flower thrips, *Frankliniella occidentalis* (Pergande), is the most economically important pest within the insect order Thysanoptera^10^. *F. occidentalis* can reduce yields of many vegetable crops (e.g. tomato, cucumber and peppers) and ornamental flowers, damage cosmetic appearance, vector plant diseases (e.g. tomato spotted wilt virus), impose quarantine risks that negatively impact trade^11^,^12^, and when present in large numbers, bite people, causing various dermal reactions^11^,^13^. Hence, its management is a matter of importance. *F. occidentalis* is endemic to western North America from Mexico to Alaska^14^. Since the late 1970s, global trade in ornamental greenhouse plants rapidly spread *F. occidentalis* around the world and now it is found on every continent but Antarctica^15^. Since the year 2000, *F. occidentalis* has rapidly invaded and become established in many provinces in almost every climate region in China^16^,^17^. In *F. occidentalis*’s native range, two habitat-specific phylogenetic lineages (ecotypes; hot/dry (HD) and cool/moist (CM) type) with allopatric distribution have been observed^18^, although these two forms might represent cryptic species as suggested by Rugman-Jones *et al.* (2010)^19^. Our previous study revealed that these two forms simultaneously established in China^20^ and that the genetic diversity of the recently introduced *F. occidentalis* populations in China is lower than that in its native range^20^. The decreased genetic diversity of *F. occidentalis* in China provides an opportunity to study the temporal genetic dynamics of recently introduced populations with lower genetic diversity. Such knowledge is crucial for the management of this species and for understanding the factors associated with invasion success.

In this study, we used a large sampling scheme including eight localities distributed in four climate regimes in China to investigate the post-established dynamics of *F. occidentalis* populations using mitochondrial and nuclear markers. We aimed to determine whether the genetic diversity, population size and the allele frequencies of the invasive populations changed and analyzed which factors might account for the temporal genetic dynamics.

## Results

**Figure 1:**
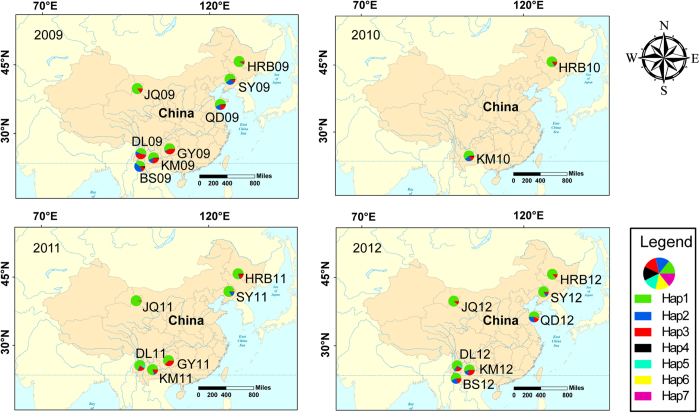
Sampling locality and mitochondrial haplotype distribution for each year (The map is made by ArcGIS 10.2 software, http://www.arcgis.com/features/).

In total, 851 *F. occidentalis* adult females were sampled from May 2009 to August 2012 (Table 1, Fig. 1). The time span represents multiple generations considering the generation number of *F. occidentalis* was 8–15 in BS, DL, KM and GY, 13-14 in QD, 1–10 in HRB and SY, 2–10 in JQ^17^,^21^. These samples were collected in eight localities, which were sampled two or more times, belonging to four different climate regions in China and characterized by discrete environmental variables (Table 1). BS, DL, KM and GY are located in the subtropical plateau monsoon climate zone with an annual temperature around 15 °C and annual precipitation around 1000 mm. QD is found in the temperate maritime monsoon climate zone with annual temperatures around 12 °C and annual precipitation around 700 mm. HRB and SY have temperate continental monsoon climates with an annual temperature around 6 °C and annual precipitation around 600 mm. JQ belongs to the temperate continental desert climate zone with annual temperatures around 7 °C and annual precipitation around 85 mm. In summary, the southwestern and northern localities were warmer than the other localities and northwestern localities were drier than the other localities.

### Comparison of genetic diversity across years within each locality

Twenty-three polymorphic sites were found in the alignment of 851 COI sequences, giving seven haplotypes (Hap1-Hap7, Table 2 and Figure S1). Two new haplotypes (Hap6 and Hap7), which were not found in previous studies, were found in Qingdao in 2012 (QD12). These two haplotypes were confirmed by performing the sequencing twice. The number of haplotypes per population and year ranged from 2 to 5, with QD12 having the highest (*N*_h_ = 5) and JQ09, JQ11, JQ12 and HRB12 having the lowest (*N*_h_ = 2). The haplotype diversity ranged from 0.053 (JQ11) to 0.667 (QD12) (Table 2). Two *F. occidentalis* forms (ecotypes or cryptic species) were simultaneously found in this study (Hap4 and Hap6 correspond to CM form and other five haplotypes correspond to HD form) (Figure S1). However, only three individuals carrying CM mitochondrial haplotypes (Hap4 and Hap6 in this study) were found in the post-2009 samples. We previously reported that hybridization occurred between the two *F. occidentalis* forms^20^ (based on the mtDNA and nuclear microsatellite data) and hypothesized that current CM individuals were hybrids. If this is the case, CM’s low frequency in all the populations and years revealed that there is no hybrid advantage for *F. occidentalis*. The levels of mtDNA variation were relatively constant across collection years within the same site (Tables 1 and 2, Fig. 1).

A total of 260 alleles were observed from 24 loci across all populations and years. The number of alleles per locus ranged from 2 (WFT24) to 28 (WFT25) with a mean of 10.8. The unbiased expected heterozygosity (u*H*_E_) per population and year ranged between 0.504 in JQ11 to 0.645 in KM12 (Table 2), and revealed no significant difference among years within the same localities except in JQ (JQ09 vs JQ11, Z = −2.571, P = 0.010). The allelic richness (*A*_R_), calculated based on the minimum number of nine individuals, ranged from 3.564 in JQ11 to 5.001 in DL09 with an average value of 4.503 across all the localities. A total of 24 pairwise tests in allelic richness (*A*_R_) among years within the same locality yielded only three significant differences (JQ09 vs JQ11, Z = −3.406, P = 0.001; JQ11 vs JQ12 Z = −3.600, P < 0.001; QD09 vs QD12, Z = −2.595, P = 0.010). Generally speaking, the genetic diversity indices among years within each locality did not change significantly.

### Genetic differentiation of the temporal samples within each locality

**Figure 2:**
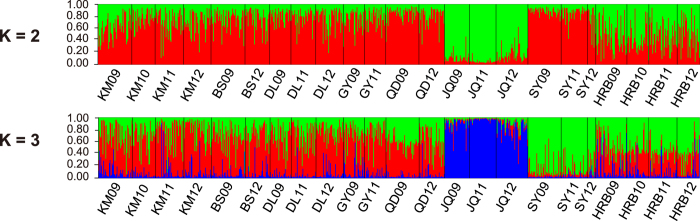
Bayesian clustering analysis of *Frankliniella occidentalis* populations. Each individual is represented by a vertical bar displaying membership coefficients to each genetic cluster. Results for K = 2 and K = 3 were shown.

The *F*_ST_ matrix showed that mtDNA haplotype frequencies among years within the same locality were not significantly different (Table S1, supporting information). Pairwise measures of microsatellite genetic distance also revealed temporal homogeneity, i.e., 18 of the 24 among-year comparisons from the same sites were not significantly differentiated (Table S2). The six among-year comparisons that revealed significant differentiation were three in JQ, two in KM and one in SY (Table S2). AMOVA did not detect any significant genetic differentiation overall between temporal groupings but it did detect genetic differentiation between geographical groupings (Table 3). Pairwise *F*_ST_ values ranged from 0.001 to 0.178 with the highest differentiation observed between JQ11 and SY12 (Table S2). This also reflected the fact that JQ was genetically isolated from other populations. As discussed in a previous study^20^, the Bayesian clustering analysis also revealed the presence of two distinct clusters (Figure S2). One hypothetical cluster includes the samples in different years in the northeastern population HRB and the samples in different years in the northwestern population JQ. However, we considered this estimate overly conservative because the posterior probability continued to increase between K = 2 and K = 3. So, when K = 3, all the samples in JQ formed a single cluster and the samples in the neighboring populations HRB and SY clustered together (Fig. 2). The K = 4 groupings did not increase likelihood values and were biologically uninterpretable. Additionally, the temporal samples for different years from the same locality always clustered together for both K = 2 and K = 3 (Fig. 2). The Bayesian clustering analysis could not distinguish the two *F. occidentalis* forms (CM and HD form) as the case in Yang *et al.* (2012)^20^. GENECLASS identified 16 individuals as potentially first-generation (F0) migrants (not listed), of which 15 migrated from the three southwestern localities (BS, DL and KM) to almost all of the other localities. In addition, we did not detect isolation by distance pattern between the eight localities based on all microsatellite loci (Z = 1843.97, R = 0.21, P = 0.14) and COI marker (Z = 3047.89, R = 0.05, P = 0.38) data.

## Discussion

We assessed changes in genetic composition of eight recently introduced populations of *F. occidentalis* in China in order to gain insight into the temporal genetic dynamics of invasive species during their post-establishment phase. The genetic composition of all but one of these populations (JQ, discussed below) was maintained over the two- to four-year study period (tens of generations) despite having lower genetic variation than the native America populations.

The present study revealed that the among-years genetic variation in the same locality was not significantly different from each other at most localities, except for the allelic richness (*A*_R_) in JQ and the unbiased expected heterozygosity (u*H*_E_) in JQ and QD. *F*_ST_ and STRUCTURE analysis also showed that most among-years comparisons from the same sites were not significantly differentiated. Furthermore, the hierarchical analysis of molecular variance (AMOVA) showed much closer genetic relationships among temporal samples from the same populations compared to samples from different populations. Generally speaking, we detected no changes in estimates of genetic diversity and temporal genetic structure in these localities over the 2–4 year periods spanning tens of generations. As discussed earlier, the genetic diversity of *F. occidentalis* in China was lower than that in its native range in America^18^,^20^. The low genetic diversity could be attributed to the genetic bottleneck and other factors such as anthropogenic controls and population fluctuations cannot be ruled out. For example, insecticide applications can lead to a decrease of the pest genetic diversity and effective population size^6^. The genetic bottleneck that we previously observed in 2009^20^ persisted in the following years. Temporal stability in the genetic composition of invasive insect species during the post-establishment phase was reported in only few cases, e.g. the invasive species oleander aphid (*Aphis nerii*) in the southern United States^22^. Founder effect was also found to persist in a lizard species over 4 years^23^. Some native species frequently exhibit temporal genetic stability. For example, populations of a riverine species of tsetse fly, *Glossina fuscipes*, were stable over 8–12 generations in Uganda^24^. Populations of rosy apple aphid, *Dysaphis plantaginea*, also showed weak temporal structure in French apple orchards^25^. On the contrary, genetic changes during the post-establishment phase were observed in a few invasive species, e.g. the whitefly *Bemisia tabaci* in China^26^ and the mosquito *Aedes japonicas* in Northeastern America^27^. Genetic parameters of introduced populations of *Rhagoletis completa* also showed greater temporal variability than native populations^28^. Similarly, significant temporal change in local genetic composition over a four-month summer cropping period was observed in *B. tabaci* populations in Queensland, Australia^29^.

However, the northwestern population JQ seems to have been temporally unstable for the genetic diversity (Table 2) and microsatellite allele frequency (Table S2) within the period studied. JQ11 had lower genetic variation than JQ09 and JQ12. Sampling bias could be a possible reason for the lower genetic diversity in JQ11 because these samples were collected in different seasons for each year (JQ11 was collected in early autumn while JQ09 and JQ12 were collected in mid-summer). However, other possibilities, such as the harsh and unstable environment in JQ (e.g. temperature differences between day and night are great and drier condition) and the small effective population size in JQ cannot be excluded.

There is evidence for a slight population genetic bottleneck as the genetic diversity was certainly reduced in the introduced populations of *F. occidentalis*^20^. The slight genetic bottleneck of *F. occidentalis* might purge deleterious alleles and thus enhance its fitness. For example, Facon *et al.* (2011)^30^ have shown that bottleneck event(s) of appropriate intensity might enable the evolution of invaders that maintain high fitness by purging deleterious alleles. In our previous study, we hypothesized that high levels of gene flow occurs in the Chinese populations. Though pest management programs such as cultural controls, biological controls, and judicious use of insecticides have been used in 26,500 hectares in Beijing, Yunnan, Shandong, Zhejiang and several other provinces in China and have partially prevented the damage and spread *of F. occidentalis*, these programs did not completely eradicate the migration and gene flow in these populations. Hence, the gain of neutral genetic variation through the gene flow might balance the loss through genetic drift.

Moreover, we cannot completely rule out the role of natural selection for the maintenance of the genetic composition of the studied populations. Local adaptation might result in the enhanced fitness and hence contributed to the maintenance of the genetic composition. It was shown that the benefits of local adaptation could balance against the inbreeding cost that could develop in part owing to the isolating effect of local adaptation itself 31. In agreement with this hypothesis, *F. occidentalis* adapted to the host plants on which they were maintained in only a few generations showed better reproductive performance than on other plants^32^. The better reproductive performance on the adapted-host plants indicates that western flower thrips have a high adaptation potential^10^,^11^ that may be due to its biological attributes. For example, *F. occidentalis* can feed on over 250 different plants in 62 different families^10^,^33^ and can produce more than 200 progeny per female per generation^10^. Moreover, they have become resistant to many pesticides^34^ and have high population growth potential^11^. The above attributes make it partially offset much of the detrimental effects of inbreeding and allows to rapidly adapt to new suitable environments^20^. The high adaptive potential of *F. occidentalis* could be an explanation for its rapid adaptation and maintenance of the genetic composition in China. Several other insects are also thought to have rapidly adapted to their specific habitats either by the stress-induced modification of the genome or by phenotypic evolution during the post-establishment period. Australia was colonized by *Drosophila buzzatii* some 600–700 generations ago. Although microsatellite DNA is usually located in the non-coding region, two of 15 microsatellites studied in this species were subjected to recent selection, one exhibiting local adaptation in different populations and the other balancing selection and these two loci may be in linkage disequilibrium with functional genes under selection^35^. Positive selection was associated with the invasion of African-derived honey bees in the New World^36^. Adaptive phenotypic changes in contemporary time scales have been reported in several species in response to rapid change in climatic regimes^37^. Urbanski *et al.* (2012)^38^ demonstrated that rapid adaptive evolution of the photoperiodic response occurred during the invasion and range expansion of the Asian tiger mosquito *Aedes albopictus* across 15 degrees of latitude in the United States. In addition, the beetle *Longitarsus jacobaeae* at high-elevation sites has adapted to the cooler conditions by life-history changes^39^. However, numerous studies have also highlighted the role of phenotypic plasticity in a population’s ability to react to a changing environment, especially for the populations with low neutral genetic diversity^40^. Thus, further studies are needed to understand the basis of phenotypic changes of *F. occidentalis* across different regions.

Our results suggest that the populations can maintain their genetic composition over a short time in their early phase of establishment despite having lower genetic diversity than their native range. However, the polymorphism level and number of the loci employed could potentially limit our ability to detect changes in genetic diversity at specific regions across the genome. Genomic data may provide more information on the changes of genetic diversity as any relevant change at the nucleotide level will not be picked up with our markers. The sensitivity for the detection of potential changes in genetic diversity and structure could also be increased with more temporal sample points and greater numbers of individuals screened. In addition, the actual levels of genetic diversity associated directly with population fitness traits or adaptive characters such as growth rate, behavior etc. cannot be directly evaluated with our approach of assaying microsatellite loci, which represent neutral genetic loci. Hence, temporal genetic stability of these populations should be interpreted cautiously since more data (more individuals and more markers) should be used to verify our conclusion. Furthermore, temporal variation of *F. occidentalis* in genetic composition was only estimated over a short time period. Whether the genetic composition and their biological and ecological characteristics of these populations will remain stable or not in the subsequent years should be monitored.

## Methods

### Mitochondrial DNA sequencing and Microsatellite genotyping

In total, 851 *F. occidentalis* adult females were sampled from May 2009 to August 2012 (Table 1, Fig. 1). The methods for sample collection, species identification were described earlier^20^,^41^. The procedure for total genomic DNA extraction and amplification of a 571-bp fragment of the mitochondrial cytochrome c oxidase subunit I gene (COI) were described in our previous study^20^.

Samples were then genotyped at 25 microsatellite loci developed for *F. occidentalis*. Ten genome-derived (WFT01-WFT08^20^, FOCC75 and FOCC125^42^) and 15 EST (expressed sequence tags)-derived polymorphic microsatellites (WFT20, WFT24, WFT25, WFT28, WFT37, WFT50, WFT51, WFT64, WFT66, WFT87, WFT98, WFT104, WFT108, WFT124 and WFT141)^43^ were used. PCR conditions were as in Brunner and Frey^42^ and Yang *et al.* (2012)^20^,^43^. PCR products were then genotyped on an Applied Biosystems 3130 Genetic Analyzer using LIZ-500 size standard. Data were collected and binned with GeneMapper v 4.0. The genotype data of the 2009 samples at ten loci (WFT01-WFT08^20^, FOCC75 and FOCC125^42^) and the COI sequence data were published in another paper^20^ and were cited in this study.

### Genetic diversity analysis

Mitochondrial DNA sequences were manually checked and assembled with CodonCode Aligner 3.6.1 (CodonCode, Dedham, MA, USA). No double peaks, frameshifts or stop codons were present in any of the sequences, suggesting that our sequences represent mtDNA and are not nuclear mitochondrial pseudogenes (numts)^44^. Multiple alignment of the resulting consensus sequences were carried out by Clustal X 2.0.11^45^. Number of haplotypes (*N*_h_), nucleotide diversity (π) and haplotype diversity (*H*_d_) for each population were assessed using DNASP v5^46^. TCS v1.21 was used to generate a haplotype network using statistical parsimony^47^.

MICRO-CHECKER 2.2.3 was used to detect genotyping errors of microsatellite data due to null alleles, stuttering, or allele dropout using 1000 randomizations^48^. Results showed that no evidence for stuttering and large allele dropout was detected in any population and year. Deviations from Hardy-Weinberg equilibrium (HWE) in all the loci of each sample and linkage disequilibrium between pairs of loci were assessed using Genepop 4.0.10^49^. Exact tests of HWE and linkage disequilibrium were conducted following Fisher’s method and GENEPOP’s default Markov chain parameters (1000 dememorizations, 100 batches and 1000 iterations). Linkage disequilibrium (LD) analysis of microsatellite markers revealed that twenty-eight of a total of 6900 tests exhibited significant LD, 18 of which were related to WFT98. WFT98 was removed from the following analysis because of concern that its linkage disequilibrium with other loci could lead to spurious results. The tests for Hardy–Weinberg equilibrium yielded only 66 significant outcomes among 575 locus/population/year combinations (data not shown). Five loci (WFT03, WFT04, WFT07, FOCC75 and WFT25) accounted for 63 of the significant outcomes. The MICRO-CHECKER analysis revealed that many of these cases were related to significant null alleles (data not shown). Consequently, we performed the following analyses both with and without these five loci to achieve unbiased and robust results. As the results were similar in both cases (data not shown), only those generated from the 24 loci data set were reported.

The genetic diversity indices such as total alleles per locus (*N*_A_), observed heterozygosity (*H*_O_), unbiased expected heterozygosity (*uH*_E_) and mean number of alleles (*N*a) were assessed using GenAlEx 6.41^50^. Allelic richness (*A*_R_) was assessed with FSTAT 2.9.3.2^51^. For each estimate of genetic variation, differences among years within each locality were tested using Wilcoxon’s signed-rank tests (for paired comparisons) with samples paired by locus.

### Genetic differentiation analysis

The degree of population differentiation based on mtDNA and microsatellite data was quantified using pairwise *F*_ST_ values in Arlequin 3.5^52^ with 10000 permutations. An analysis of molecular variation (AMOVA) implemented in Arlequin 3.5 was used to test the hierarchic genetic structure of the populations. Significance for AMOVA analysis was ascertained using 10000 permutations. Sequential Bonferroni correction was used for tests involving multiple comparisons^53^.

We then used STRUCTURE 2.3.3^54^ and its non-spatial algorithm to further assess the degree of population differentiation within and between the eight localities using microsatellite data. The correlated allele frequencies and admixed model were applied with a burn-in period of 300000 and 500000 MCMC iterations after burn-in. We specified an initial range of potential genotype clusters (K) from 1 to 10 with 10 runs. To estimate the most likely K value we utilized both the log likelihood (lnPr(X/K)) method as recommended by Pritchard *et al.* (2000)^54^ and the *Δ*K statistic of Evanno *et al.* (2005)^55^. Detection of first generation migrants for each population was performed in GENECLASS 2 using L-home likelihood computation^56^. Mantel test for isolation by distance, as revealed by a correlation between pairwise linearized genetic and log-geographic distances (Euclidean), was performed using IBDWS 3.16^57^.

## Additional Information

**How to cite this article**: Yang, X.-M. *et al.* Temporal Genetic Dynamics of an Invasive Species, *Frankliniella occidentalis* (Pergande), in an Early Phase of Establishment. *Sci. Rep.* **5**, 11877; doi: 10.1038/srep11877 (2015).

## Supplementary Material

Supplementary Information

## Figures and Tables

**Table 1:** Collection information and three environmental variables for samples of *Frankliniella occidentalis* used in this study.

Code	Sampling dates	Nb samples	Location	Coordinates	Host	T(°C)	Precipitation (mm)	RH
KM09	Aug. 10-11, 2009	48	Kunming	24°42′43.91″N, 102°43′07.76″E	*Dianthus caryophyllus*; *Trifolium* L.	15.07	988.46	71.69
KM10	Jul. 3, 2010	33						
KM11	Jul. 25, 2011	40						
KM12	Aug. 24, 2012	39						
BS09	Aug. 5, 2009	48	Baoshan	25°10′24.55″N, 99°13′12.53″E	*Solanum melongena L.; Brassica campestris L.*	15.96	978.21	73.39
BS12	Aug. 21, 2012	35						
DL09	Aug. 7, 2009	30	Dali	25°36′17.49″N, 100°14′49.75″E	*Trifolium* L.; *Nicandra physalodes*; *Canna indica* L.; *Rosa chinensis*	15.01	1063.29	67.71
DL11	Jul. 30, 2011	35						
DL12	Aug. 20, 2012	39						
GY09	Apr. 25, 2009	30	Guiyang	26°39′46.08″N, 106°48′57.38″E	*Petunia hybrida* Vilm; *Cucurbita moschata*; *Cucurbita pepo* L.	15.13	1114.25	77.32
GY11	Jul. 26, 2011	30						
QD09	Jun. 1-2, 2009	47	Qingdao	36°19′10.29″N, 120°23′32.18″E	*Trifolium* L.; *Rosa chinensis*	12.67	716.52	71.06
QD12	May 31, 2012	36						
JQ09	Jul. 16-17, 2009	35	Jiuquan	39°46′42.82″N, 98°30′21.88″E	*Tagetes erecta* L.	7.50	85.51	47.00
JQ11	Sep. 5, 2011	38						
JQ12	Jul. 21, 2012	45						
SY09	26 Aug. 2009	47	Shenyang	41°49′49.10″N, 123°34′09.65″E	*Fuchsia hybrida* Voss; *Petunia hybrida* Vilm	8.16	710.61	63.95
SY11	Aug. 30, 2011	37						
SY12	Jul. 13, 2012	12						
HRB09	Aug. 23, 2009	44	Harbin	45°44′30.54″N, 126°37′59.84″E	*Tagetes erecta* L.; *Hosta ventricosa* (Salisb.) Stearn	4.25	526.49	65.32
HRB10	Jul. 23, 2010	31						
HRB11	Aug. 25, 2011	40						
HRB12	Jul. 5, 2012	32						

Nb samples, number of samples; T, annual mean temperature; RH, relative humidity.

**Table 2:** Genetic diversity indices calculated using COI gene and 24 microsatellites and haplotype distribution in Chinese populations of *Frankliniella occidentalis*.

Pop	mtDNA										Microsatellite				
	*N*_h_	Hap1	Hap2	Hap3	Hap4	Hap5	Hap6	Hap7	*H*_d_	π (±SD)	*N*a	*H*_E_	u*H*_E_	*H*o	*A*_R_
KM09	4	27	7	13	1				0.601 (0.053)	0.00254 (0.00131)	7.125	0.611	0.617	0.506	4.717
KM10	4	21	7	5	1				0.570(0.077)	0.00300(0.00180)	7.042	0.616	0.626	0.542	4.925
KM11	3	32	2	6					0.344(0.086)	0.00063(0.00017)	6.583	0.631	0.639	0.542	4.726
KM12	3	22	6	12					0.600(0.052)	0.00121(0.00015)	7.042	0.636	0.645	0.526	4.953
BS09	4	19	21	6	2				0.648 (0.037)	0.00399 (0.00175)	6.833	0.606	0.613	0.545	4.597
BS12	3	21	9	7					0.599(0.059)	0.00121(0.00016)	6.250	0.577	0.586	0.461	4.532
DL09	4	13	3	13		1			0.634 (0.049)	0.00133 (0.00018)	6.792	0.617	0.628	0.518	5.001
DL11	4	24	1	7		3			0.496(0.086)	0.00096(0.00020)	6.458	0.615	0.624	0.553	4.667
DL12	4	25	3	8		3			0.549(0.078)	0.00110(0.00019)	7.000	0.619	0.627	0.574	4.825
GY09	3	17	1	12					0.536 (0.048)	0.00099 (0.00012)	5.958	0.592	0.602	0.530	4.466
GY11	2	19		11					0.480(0.052)	0.00084(0.00009)	6.292	0.598	0.608	0.540	4.506
QD09	4	27	9	1	1				0.600 (0.057)	0.00257 (0.00133)	6.292	0.602	0.608	0.533	4.401
QD12	5	18	9	7			1	1	0.667(0.055)	0.00309(0.00162)	6.458	0.611	0.620	0.542	4.707
JQ09	2	3		5					0.252 (0.085)	0.00044 (0.00015)	5.917	0.585	0.594	0.506	4.454
JQ11	2	37		1					0.053(0.049)	0.00009(0.00009)	4.708	0.497	0.504	0.438	3.564
JQ12	2	4		5					0.202(0.073)	0.00035(0.00013)	6.208	0.571	0.578	0.496	4.304
SY09	4	29	13	4	1				0.547 (0.060)	0.00241 (0.00133)	5.667	0.562	0.568	0.476	4.063
SY11	3	31	5	1					0.287(0.088)	0.00052(0.00016)	5.583	0.555	0.562	0.470	4.118
SY12	3	1	1	1					0.318(0.164)	0.00058(0.00032)	4.292	0.504	0.527	0.462	3.964
HRB09	3	4	1	3					0.172 (0.074)	0.00031 (0.00013)	6.583	0.595	0.602	0.535	4.525
HRB10	3	26	1	4					0.288(0.097)	0.00052(0.00018)	6.042	0.563	0.573	0.503	4.396
HRB11	4	32	1	6	1				0.345(0.087)	0.00221(0.00156)	6.875	0.596	0.603	0.522	4.670
HRB12	2	28		4					0.226(0.088)	0.00040(0.00015)	6.083	0.588	0.598	0.494	4.490
Total		588	100	151	7	7	1	1							
Mean									0.435		6.264	0.589	0.598	0.514	4.503

*N*_h_ , number of haplotypes; *H*_d _, haplotype diversity; π, nucleotide diversity; *N*a , number of alleles; *H*_E _, expected heterozygosity; *uH*_E_ , unbiased expected heterozygosity; *H*_O_ , observed heterozygosity; *A*_R_ , allelic richness.

**Table 3:** Results of AMOVA test on mitochondrial and microsatellite markers.

Groups	Source of variation	mtDNA		Microsatellite	
		% variation	Fixation indices	% variation	Fixation indices
Four temporal groups	Among groups	−0.053	FCT = −0.001 (P = 0.494)	−0.333	FCT = −0.003 (P = 0.999)
	Among pops within groups	4.426	FSC = 0.044 (P < 0.001)	4.761	FSC = 0.047 (P < 0.001)
	Within populations	95.627	FST = 0.044 (P < 0.001)	95.573	FST = 0.044 (P < 0.001)
Seven location groups	Among groups	4.608	FCT = 0.046 (P < 0.001)	2.866	FCT = 0.029 (P < 0.001)
	Among pops within groups	0.197	FSC = 0.002 P = 0.191)	1.906	FSC = 0.020 (P < 0.001)
	Within populations	95.196	FST = 0.048 (P < 0.001)	95.228	FST = 0.048 (P < 0.001)
