# Supplementary material for: Temporal Genetic Dynamics of an Invasive Species, Frankliniella occidentalis (Pergande), in an Early Phase of Establishment
Source: Sci Rep. 2015 Jul 3;5:11877. doi: 10.1038/srep11877 (PMC4490395; doi:10.1038/srep11877)
Supplement: Supplementary Information [file srep11877-s1.pdf]

Temporal Genetic Dynamics of an Invasive Species, *Frankliniella occidentalis* (Pergande), in an Early Phase of Establishment

Xian-Ming Yang, Heng Lou, Jing-Tao Sun, Yi-Ming Zhu, Xiao-Feng Xue and Xiao-Yue Hong*

(Initials of author names: Yang XM, Lou H, Sun JT, Xue XF and Hong XY)

Department of Entomology, Nanjing Agricultural University, Nanjing, Jiangsu 210095, China

*Corresponding author.

Xiao-Yue Hong

Department of Entomology

Nanjing Agricultural University

Nanjing, Jiangsu 210095

CHINA

Tel & Fax: 0086-25-84395339

E-mail address: xyhong@njau.edu.cn (X.-Y. Hong).

Table S1. Pairwise F_{ST} values based on COI data. Significant values (P < 0.00091) after Bonferroni correction are indicated in bold.

	KM09	KM10	KM11	KM12	BS09	BS12	DL09	DL11	DL12	GY09	GY11	QD09	QD12	JQ09	JQ11	JQ12	SY09	SY11	SY12	HRB09	HRB10	HRB11
KM10	-0.01																					
KM11	0.01	0.02																				
KM12	-0.01	0.02	0.04																			
BS09	0.04	0.00	**0.10**	0.07																		
BS12	-0.01	-0.01	0.04	0.00	0.03																	
DL09	0.00	0.07	0.11	0.00	0.10	0.06																
DL11	0.01	0.03	0.00	0.03	**0.10**	0.05	0.06															
DL12	0.00	0.02	-0.01	0.01	0.08	0.02	0.05	-0.02														
GY09	0.01	0.07	0.10	0.01	**0.12**	0.08	-0.02	0.05	0.04													
GY11	0.01	0.07	0.08	0.01	**0.13**	0.09	-0.01	0.04	0.04	-0.03												
QD09	-0.02	-0.02	0.01	-0.01	0.02	-0.02	0.03	0.01	0.00	0.03	0.03											
QD12	-0.01	-0.02	0.03	0.00	0.00	-0.02	0.04	0.03	0.02	0.05	0.06	-0.02										
JQ09	0.02	0.03	-0.02	0.07	**0.12**	0.08	0.14	0.00	0.01	0.12	0.10	0.03	0.05									
JQ11	0.08	0.06	0.06	**0.20**	**0.14**	**0.17**	**0.31**	0.09	0.09	**0.32**	**0.31**	0.07	**0.08**	0.06								
JQ12	0.04	0.05	-0.01	0.11	**0.13**	0.11	0.20	0.02	0.03	0.18	0.15	0.04	0.06	-0.02	0.03							
SY09	0.02	-0.02	0.05	0.04	0.00	0.00	0.10	0.06	0.04	**0.11**	**0.12**	0.00	-0.02	0.07	0.08	**0.08**						
SY11	0.05	0.00	0.04	0.12	0.07	0.05	**0.23**	0.08	0.06	**0.25**	**0.24**	0.02	0.03	0.08	0.07	0.07	0.01					
SY12	-0.01	-0.03	-0.04	0.03	0.03	0.00	0.12	-0.01	-0.02	0.13	0.12	-0.02	-0.02	-0.02	0.03	-0.02	-0.01	-0.04				
HRB09	0.05	0.04	0.00	0.13	**0.12**	0.11	**0.24**	0.05	0.05	0.23	0.21	0.04	0.06	0.00	-0.01	-0.01	0.07	0.04	-0.04			
HRB10	0.01	0.02	-0.03	0.06	0.09	0.05	0.13	0.00	0.00	0.12	0.10	0.01	0.03	-0.03	0.04	-0.02	0.05	0.04	-0.04	-0.01		
HRB11	0.00	0.00	-0.01	0.03	0.07	0.03	0.06	0.00	0.00	0.05	0.03	0.00	0.01	-0.01	0.02	0.00	0.03	0.02	-0.04	0.00	-0.01	
HRB12	0.03	0.03	-0.02	0.08	**0.11**	0.09	0.16	0.01	0.01	0.14	0.12	0.03	0.05	-0.03	0.04	-0.03	0.06	0.07	-0.02	-0.01	-0.03	-0.01

Table S2. Pairwise F_{ST} values based on 24 microsatellites. Significant values (P < 0.00091) after Bonferroni correction are indicated in bold.

	KM09	KM10	KM11	KM12	BS09	BS12	DL09	DL11	DL12	GY09	GY11	QD09	QD12	JQ09	JQ11	JQ12	SY09	SY11	SY12	HRB09	HRB10	HRB11
KM10	0.009																					
KM11	**0.016**	0.005																				
KM12	**0.014**	0.003	0.003																			
BS09	**0.019**	0.009	**0.012**	0.008																		
BS12	**0.019**	0.004	0.004	0.008	0.001																	
DL09	0.010	0.000	0.005	0.000	0.007	0.002																
DL11	**0.021**	0.008	0.010	0.009	0.012	0.011	0.007															
DL12	**0.019**	0.003	0.004	0.004	**0.012**	0.001	0.002	0.004														
GY09	**0.036**	**0.024**	**0.018**	**0.021**	**0.030**	**0.018**	**0.021**	**0.031**	**0.027**													
GY11	**0.026**	0.013	0.011	0.012	**0.017**	0.006	0.007	**0.017**	**0.015**	0.006												
QD09	**0.034**	**0.022**	**0.028**	**0.029**	**0.024**	**0.025**	**0.018**	**0.037**	**0.032**	**0.038**	**0.030**											
QD12	**0.022**	0.010	**0.020**	**0.017**	**0.015**	0.013	0.010	**0.024**	**0.020**	**0.028**	0.015	0.006										
JQ09	**0.056**	**0.059**	**0.064**	**0.055**	**0.064**	**0.070**	**0.059**	**0.074**	**0.074**	**0.065**	**0.064**	**0.086**	**0.059**									
JQ11	**0.107**	**0.114**	**0.105**	**0.119**	**0.120**	**0.120**	**0.123**	**0.135**	**0.122**	**0.125**	**0.120**	**0.145**	**0.119**	**0.075**								
JQ12	**0.051**	**0.055**	**0.060**	**0.047**	**0.055**	**0.063**	**0.057**	**0.065**	**0.064**	**0.068**	**0.063**	**0.079**	**0.056**	**0.037**	**0.081**							
SY09	**0.042**	**0.036**	**0.045**	**0.040**	**0.042**	**0.046**	**0.031**	**0.042**	**0.045**	**0.055**	**0.050**	**0.046**	**0.040**	**0.102**	**0.168**	**0.093**						
SY11	**0.044**	**0.033**	**0.043**	**0.047**	**0.042**	**0.047**	**0.033**	**0.044**	**0.047**	**0.055**	**0.049**	**0.038**	**0.035**	**0.105**	**0.168**	**0.102**	0.010					
SY12	**0.042**	**0.036**	**0.043**	**0.044**	**0.050**	**0.053**	**0.029**	**0.040**	**0.041**	**0.048**	**0.040**	**0.049**	**0.045**	**0.099**	**0.178**	**0.092**	**0.038**	0.030				
HRB09	0.011	**0.018**	**0.016**	**0.018**	**0.019**	**0.023**	0.015	**0.020**	**0.025**	**0.032**	**0.028**	**0.041**	**0.026**	**0.052**	**0.106**	**0.052**	**0.043**	**0.046**	**0.041**			
HRB10	**0.023**	**0.026**	**0.027**	**0.030**	**0.032**	**0.026**	**0.024**	**0.025**	**0.029**	**0.041**	**0.032**	**0.050**	**0.032**	**0.061**	**0.107**	**0.067**	**0.058**	**0.059**	**0.049**	0.008		
HRB11	**0.022**	**0.023**	**0.019**	**0.023**	**0.029**	**0.028**	**0.019**	**0.021**	**0.024**	**0.037**	**0.031**	**0.046**	**0.031**	**0.064**	**0.098**	**0.066**	**0.044**	**0.048**	**0.043**	0.008	0.002	
HRB12	**0.026**	**0.027**	**0.022**	**0.029**	**0.030**	**0.028**	**0.022**	**0.026**	**0.031**	**0.044**	**0.036**	**0.049**	**0.034**	**0.061**	**0.103**	**0.063**	**0.049**	**0.054**	**0.049**	0.010	0.006	0.006

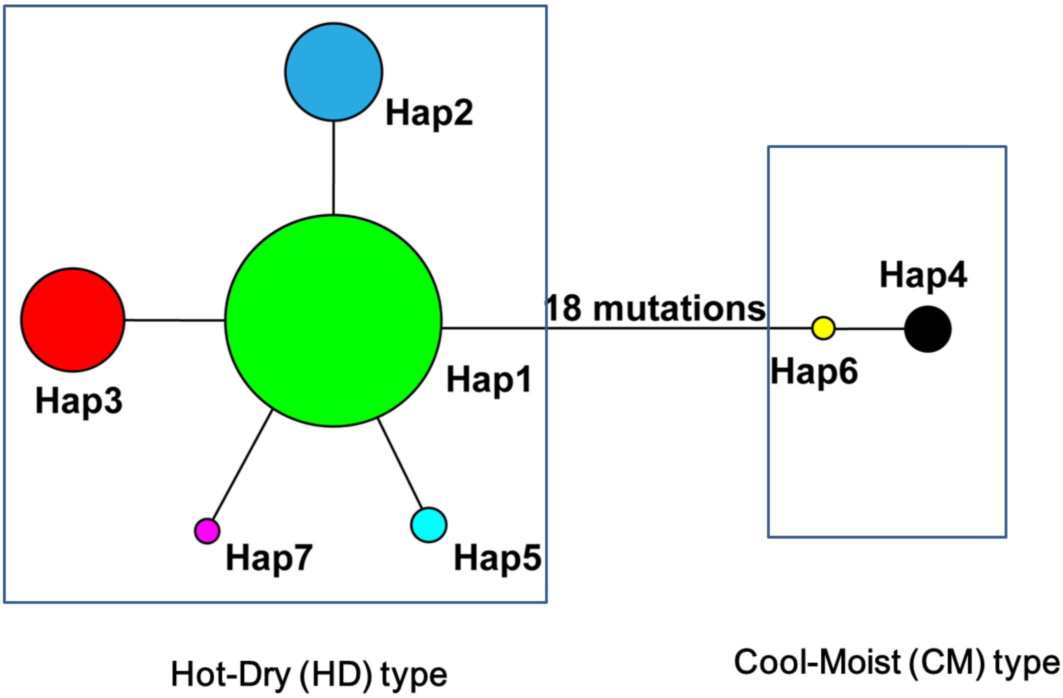

Figure S1 Haplotype network based on mitochondrial COI sequence. Each line between circles represents one mutational event unless otherwise stated.

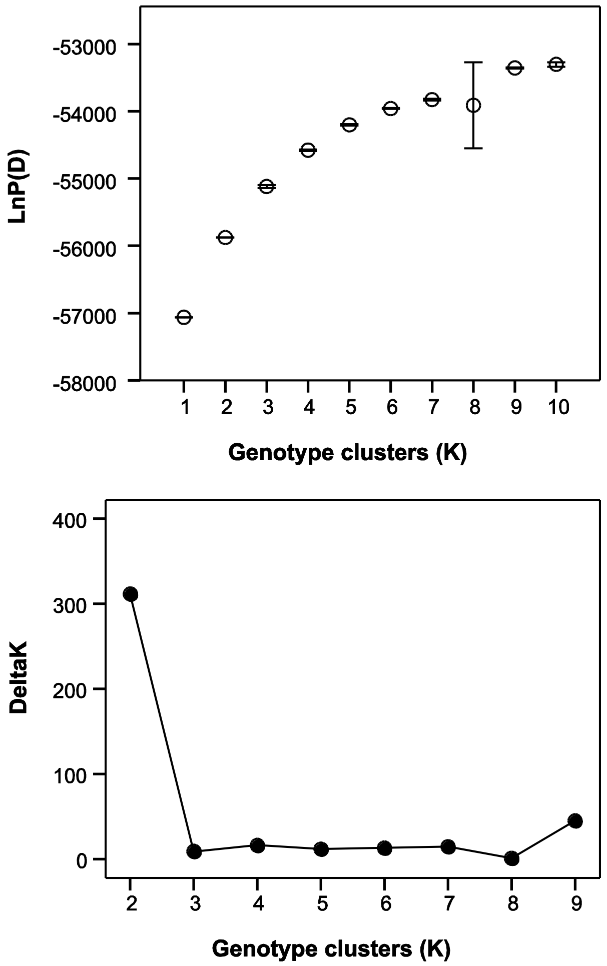

Figure S2 Inference of the number of genetic clusters (K) from STRUCTURE simulations for Chinese *Frankliniella occidentalis* populations. The likelihood of the data given K {ln P(D)} and ΔK are plotted against the number of genetic clusters (K). Error bars represent standard deviations over ten runs.
